# Far-Field and Non-Intrusive Optical Mapping of Nanoscale Structures

**DOI:** 10.3390/nano12132274

**Published:** 2022-07-01

**Authors:** Guorong Guan, Aiqin Zhang, Xiangsheng Xie, Yan Meng, Weihua Zhang, Jianying Zhou, Haowen Liang

**Affiliations:** 1 State Key Laboratory of Optoelectronic Materials and Technologies, School of Physics, Sun Yat-Sen University, Guangzhou 510275, China; guangr@mail2.sysu.edu.cn (G.G.); aiqin_zhang@126.com (A.Z.); 2 School of Electronics and Information Technology, Sun Yat-Sen University, Guangzhou 510275, China; 3 Department of Physics, College of Science, Shantou University, Shantou 515063, China; xxs@stu.edu.cn; 4 State Key Laboratory of Analytical Chemistry for Life Science, MOE Key Laboratory of Intelligent Optical Sensing and Manipulation, Jiangsu Key Laboratory of Artificial Functional Materials, College of Engineering and Applied Sciences, Nanjing University, Nanjing 210093, China; skywalker16@126.com (Y.M.); zwh@nju.edu.cn (W.Z.)

**Keywords:** far-field, non-intrusive, local density of state, super-resolution, nanostructure

## Abstract

Far-field high-density optics storage and readout involve the interaction of a sub-100 nm beam profile laser to store and retrieve data with nanostructure media. Hence, understanding the light–matter interaction responding in the far-field in such a small scale is essential for effective optical information processing. We present a theoretical analysis and an experimental study for far-field and non-intrusive optical mapping of nanostructures. By a comprehensive analytical derivation for interaction between the modulated light and the target in a confocal laser scanning microscopy (CLSM) configuration, it is found that the CLSM probes the local density of states (LDOSs) in the far field rather than the sample geometric morphology. With a radially polarized (RP) light for illumination, the far-field mapping of LDOS at the optical resolution down to 74 nm is obtained. In addition, it is experimentally verified that the target morphology is mapped only when the far-field mapping of LDOS coincides with the geometric morphology, while light may be blocked from entering the nanostructures medium with weak or missing LDOS, hence invalidating high-density optical information storage and retrieval. In this scenario, nanosphere gaps as small as 33 nm are clearly observed. We further discuss the characterization for far-field and non-intrusive interaction with nanostructures of different geometric morphology and compare them with those obtainable with the projection of near-field LDOS and scanning electronic microscopic results.

## 1. Introduction

With the rapid development of big data and artificial intelligence, emerging information technology compels an increasing demand for data information storage [1,2,3,4], while conventional magnetization-based methods suffer from the bottleneck of the slow speed of write-in and read-out, large volume, and comparatively low storage capacity. In comparison, optical storage is expected to be able to crack the hard nut [2,3]. Since the storage media are designed by complex photonic or plasmonic systems at subwavelength scales, it is inherently restricted by the optical diffraction limit, and it requires the optical system to achieve super-resolution beyond such barriers [4,5,6]. Therefore, researchers have endeavored to develop super-resolution optical storage techniques in both near-field and far-field. 

The most direct super-resolution techniques can be achieved with near-field techniques [7,8,9,10], which collect and reconstruct the evanescent field scattered or irradiated by the targets. Higher spatial frequencies could then be recovered; thus, higher optical storage density can be achieved [11]. Despite the fact that near-field techniques could provide an effective solution, the confined propagation distance of evanescent waves requires the excitation source or detection probe to be physically close to the target, hence disturbing the physical property of the target on top of the inherent probe sensitive mapping. In contrast, far-field super-resolution techniques that can reduce the full width at half maximum (FWHM) of the intensity distribution of focal spot in the far-field are able to avoid such physical disturbance. The two-photon absorption (TPA)-based process [12,13,14] and stimulated emission depletion (STED) fluorescence-inspired approaches [15,16] are typical ways to achieve this goal. However, the TPA process relies on a non-linear interaction between light and the recording medium, which highly depends on the materials, while the STED process has to use fluorescence functional groups as the labels, which is chemically intrusive to the target, and only the position information can be recorded. Therefore, there is a necessity for further development of far-field and non-intrusive super-resolution optical storage methods.

It is proposed that vectorial-field-modulated light is effective in generating a focal spot with an FWHM beyond the diffraction limit [17,18,19,20,21], which can be implemented into a confocal laser scanning microscopic (CLSM) configuration to achieve label-free super-resolution imaging [22,23,24,25,26] and storage [27,28]. While researchers endeavor to narrow the focal spot, a fundamental question remains if the light may be blocked from entering the nanostructures medium no matter how small the interacting light field is, as the optical properties rather than the actual morphology of complex photonics or plasmonic structures do really support the concentrated excitations. 

In this work, we present a theoretical analysis and an experimental study to answer what optical properties of the complex nanostructures are probed by the far-field and non-intrusive super-resolution techniques. By a comprehensive analytical derivation for interaction between the modulated light and the target in a CLSM configuration, it is found that the CLSM does probe the local density of states (LDOSs) in the far-field [29,30] rather than the sample geometric morphology. As the LDOS characterizes the overall effect of light–matter interaction in structured environments [31,32,33,34,35], it reflects the surrounding circumstance, the supporting substrate, and adjacent nanostructures will generate the synthetically optical responses on the target, which will intrinsically make the mapping information different from the geometric morphology of the target. This optical property that can usually be probed only by the near-field techniques [36,37,38,39,40] or electronic excited luminescence [41,42] can also be probed in the far-field when the optical resolution of CLSM reaches sub-100 nm. To verify this point of view, a CLSM with radially polarized (RP) light for illumination is built, and the far-field mapping of LDOS is successfully obtained with the optical resolution down to 74 nm. The experiments verify that the target morphology can be correctly mapped only when the far-field mapping of LDOS coincides with the geometric morphology, while light may be blocked from entering the nanostructures medium with weak or missing LDOS, hence invalidating high-density optical information storage and retrieval. We further discuss the characterization for far-field and non-intrusive interaction with nanostructures of different geometric morphology and compare them with those obtained from the projection of near-field LDOS and scanning electronic microscopic results. We believe the unprecedented findings in this work well describe the intrinsic physics of far-field and non-intrusive techniques to interact with complex photonic and plasmonic nanostructures, and it is also of great significance to guide the design of optical storage and retrieval systems to be more effective by avoiding the blocking of light induced by LDOS.

## 2. Theory

### 2.1. Resolving Ability of the Far-Field Non-Intrusive CLSM

One of the finest means for far-field non-intrusive optical mapping is through CLSM. Sharper focus can be achieved in this configuration with spatially modulated RP incident beams. By harnessing both excitation and detection PSFs, the lateral resolution of RP-beam-assisted CLSM can reach about 1/5 λ. Therefore, RP-beam-assisted CLSM is utilized to achieve an optical resolution beyond sub-100 nm for visible light illumination.

**Figure 1:**
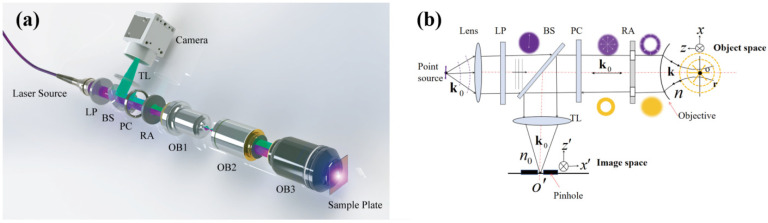
(**a**) A schematic diagram and (**b**) the optical path of the proposed far-field and non-intrusive CLSM system. The purple illustrations above the excitation system and yellow ones under the detection system, respectively, denote the morphology of optical field during the modulation processes. LP: linear polarizer; BS: beam splitter; PC: polarization converter; RA: ring aperture; OB: objective; TL: tube lens.

**Figure 2:**
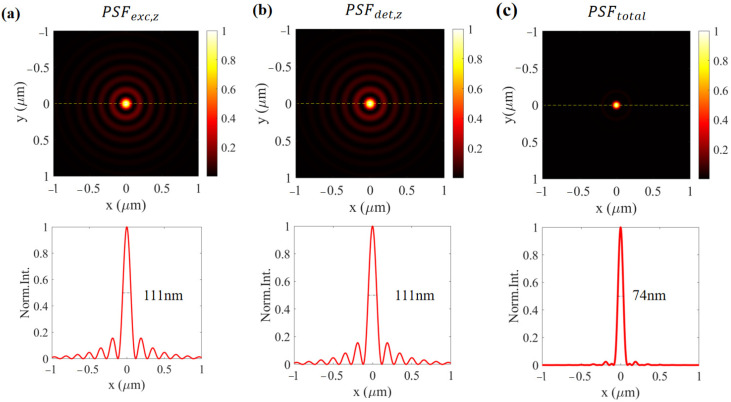
(**a**) The longitudinal component of PSF for excitation system, (**b**) the intensity of the detection PSF, and (**c**) the total PSF for the whole CLSM. The magnification of the objective in the CLSM is set as 100×.

The scheme and the optical path of the proposed far-field non-intrusive CLSM are shown in Figure 1a and Figure 1b based on the reflective configuration, respectively. The same configuration can simultaneously perform the excitation process (see Figure S1 in Supplementary Information) and the detection process (see Figure S2 in Supplementary Information). The linearly polarized beam with a wavelength of 405 nm is used as the incident light in the system. For the excitation process, the incident beam is converted into an RP beam by a polarization converter; then, the annular aperture further filters out the lower spatial frequencies of the RP beam so that the oil-immersion objective with a numerical aperture (NA) as high as 1.4 can achieve tight focusing of the high spatial frequencies light. The excitation field can then be derived (see Supplementary Information Section I for detailed derivation) as:

(1) $$E_{\mathrm{exc}}(r)=\frac{\mathrm{if}e^{-ikf}}{2\pi }{ℱ}^{-1}\left\{\frac{E_{0}(k_{x},k_{y})P(k_{x},k_{y})}{k\sqrt{\left(k_{x}^{2}+k_{y}^{2}\right)}}\sqrt{\frac{k_{z}}{nk}}\left[\begin{array}{l} k_{x}k_{z}\\ k_{y}k_{z}\\ -(k_{x}^{2}+k_{y}^{2}) \end{array}\right]\frac{e^{ik_{z}z}}{k_{z}}\right\},\;$$

where *f* is the focal length of the objective, *n* is the refractive index of the immersion oil, *k* = *nk*_0_ is the wave vector in the object plane, *z* is the longitudinal position on the object plane, *E*_0_ (*k_x_*, *k_y_*) describes the amplitude of incident RP field, and *P*(*k_x_*, *k_y_*) is the apodization function determined by both the annular aperture and objective. It has been thoroughly studied that the longitudinal component **E***_exc,z_* is much stronger than the transversal component of the tight focusing RP beam, which can be approximated as the excitation field. As shown in Figure 2a, the full-width-at-half-maximum (FWHM) intensity of the focusing field of **E***_exc,z_*, also known as the excitation PSF, is 111 nm.

To obtain the final resolution for the system, the detection of PSF should also be taken into consideration. A dipole is used in this model to characterize the detection of PSF, and its reflective information is collected in the detection process and modulated by the same optical elements as those used for excitation. The detection field is reflected by the beam splitter, coupled by the tube lens, filtered by a pinhole, and recorded on the image plane. The detection PSF can then be derived in the form of Green’s function (see Supplementary Information Section I for detailed derivation and clarification of each parameter):

(2) $${\overset{↔}{G}}_{\det}(x^{\prime },y^{\prime },z^{\prime })={\overset{↔}{G}}_{\det}(xM,yM,zM^{2}/n)=A_{\det}^{0}\cdot {ℱ}^{-1}\left\{T_{\det}(k_{x},k_{y},k_{z})\right\}$$

where *M* denotes the magnification of the detection system, (*x’*, *y’*, *z’*) is the coordinate in the image plane, $A_{\det}^{0}$ is a complex coefficient, and **T***_det_* denotes the optical transfer function (OTF) of the dipole. To have an insight of the oscillating properties of the dipole, **T***_det_* can be decomposed as **T**^x^, **T**^y^, and **T**^z^, which describes the dipole orientating along x-, y-, and z-direction, respectively (see Supplementary Information Section I for detailed derivation):

(3) $$T^{x}\propto \left[\begin{array}{l} \left(k_{x}^{3}k_{z}^{2}+k_{x}k_{y}^{2}k_{z}^{2}+k_{x}{\left(k_{x}^{2}+k_{y}^{2}\right)}^{2}\right)/k^{3}\\ \left(-k_{y}^{3}-k_{x}^{2}k_{y}\right)/k_{z}\\ 0 \end{array}\right]$$

(4) $$T^{y}\propto \left[\begin{array}{l} \left(k_{y}^{3}k_{z}^{2}+k_{x}^{2}k_{y}k_{z}^{2}+k_{y}{\left(k_{x}^{2}+k_{y}^{2}\right)}^{2}\right)/k^{3}\\ \left(k_{x}^{3}+k_{x}k_{y}^{2}\right)/k_{z}\\ 0 \end{array}\right]$$

(5) $$T^{z}\propto \left[\begin{array}{l} \left[-k_{x}^{2}k_{z}(k_{x}^{2}+k_{y}^{2})-k_{y}^{2}k_{z}(k_{x}^{2}+k_{y}^{2})-{\left(k_{x}^{2}+k_{y}^{2}\right)}^{3}/k_{z}\right]/k^{3}\\ 0\\ 0 \end{array}\right]$$

The matrices above show that the x- and y-oriented dipoles only have x- and y-components, while the z-oriented dipole only has an x-component. With $E_{\det}(x^{\prime },y^{\prime },z^{\prime })=\frac{\omega^{2}}{\varepsilon_{0}c^{2}}{\overset{↔}{G}}_{\det}(x^{\prime },y^{\prime },z^{\prime })\cdot \mu_{0}$, where **μ**_0_ denotes the dipole moment, each component of the electric field of the orientating dipoles is depicted in Figure S4. It can be seen that only the x-component of the z-oriented dipole has a non-zero field in the center; therefore, filtered by a small pinhole, the detected signal mainly originates from the z-oriented dipole that PSF*_det,z_* can be approximately taken as the detection of PSF, as shown in Figure 2b. As the total PSF of the far-field non-intrusive CLSM is determined by both the excitation of PSF and the detection of PSF, the total PSF of the system is illustrated in Figure 2c, with an FWHM of 74 nm, which is well beyond sub-100 nm.

### 2.2. The Influence of Light Matter Interaction to Optical Nanoscale Mapping

As previously mentioned, the longitudinal component **E***_exc,z_* is much stronger than the transversal component in the excitation field of the tight-focusing RP beam within its FWHM near the optic axis; hence, the contribution of the polarization induced by the transverse field can be neglected. A nano-object is further assumed to be constituted by an assembly of dipoles. Therefore, the polarization of each excited dipole **P**_0_ can be denoted as:

(6) $$P_{0}(r_{j})={\left[0,\;0,\;\alpha (r_{j})E_{exc,z}(r_{j})\right]}^{T}$$

where **α**(**r***_j_*) is the electric susceptibility at a certain position of **r***_j_*. The radiation from this dipole is:

(7) $$Ι(r_{j})=-\frac{1}{2}\int_{V}dr\;Re\;\left[J(r)\cdot E(r)\right]\;$$

where $J(r)=-i\omega P_{0}(r_{j})\delta (r-r_{j})$ is the current density of the induced dipole at **r***_j_*, and the actual electric field of the induced dipole is $E(r)=\overset{↔}{S}(r,r_{j})\cdot P_{0}(r_{j})$. $\overset{↔}{S}(r,r_{j})$ is the field susceptibility of the whole system [40,43], which is described by $\overset{↔}{S}(r,r_{j})={\overset{↔}{G}}_{\det}(r,r_{j})+{\overset{↔}{G}}_{s}(r,r_{j})$, where ${\overset{↔}{G}}_{\det}(r,r_{j})$ is the detection of Green’s function in free space, and ${\overset{↔}{G}}_{s}(r,r_{j})$ denotes the interruption of the detection of PSF induced by the extra scattered field. As a result, the radiation can be described as (see Supplementary Information Section I for detailed derivation):

(8) I(rj)=ω2[α(rj)Eexc,z(rj)]2Im{Szz(rj,rj)} 

where *S_zz_* is the z-component of the field susceptibility $\overset{↔}{S}$.

**Figure 3:**
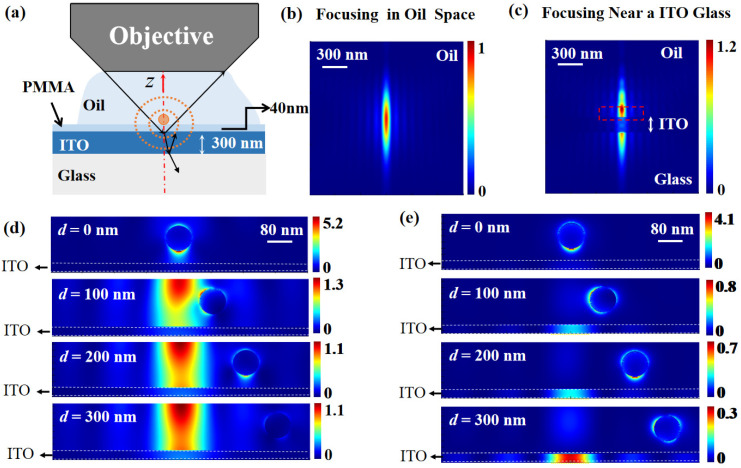
(**a**) Schematic show of light matter interaction with the excitation field, the nanosphere, and its surroundings. z denotes the coordinate direction. (**b**,**c**) show the simulated focusing field in immersion oil without and with the substrate, respectively. (**d**) The total field of the nanosphere with different distances *d* away from the center of the focus. The bottom of the nanosphere and the ITO layer are separated by a 40 nm PMMA layer. (**e**) The corresponding scattered fields by removing the excitation field.

It is worth noting that, even though the far-field non-intrusive CLSM is capable of achieving an optical resolution of sub-100 nm, the interaction of the optical field with the object and its surroundings will further impose an influence on the optical nanoscale mapping. For the excitation, the reflection field on the interface of the supporting layer will be superimposed on the excitation field. For the detection, the reflective field will also interact with the nano-objects, with which extra scattered fields will inevitably occur to interrupt the detection field. As a result, if an ensemble of nano-objects is considered, the local field is the superposition of the incident radiation and all the partial fields scattered by the surrounding, as shown in Figure 3a. Therefore, it turns out that the dipoles are interacting with each other, and each dipole is dependent on all other neighboring dipoles and structures. Such a process can be described by the volume-integral method [44], which cannot be ignored for sub-100 nm far-field non-intrusive microscopy. To intuitively demonstrate this phenomenon, the excitation field derived from the previous part is imported into a finite difference time domain software (Anasys Lumerical 2022R1, Vancouver, BC, Canada) for simulation. Detailed simulation conditions can be referred in to in the Supplementary Information Section II (Figure S3).

In the simulation, the immersion oil and the substrate, consisting of a 40 nm thick polymethylmethacrylate (PMMA) layer, an indium-tin-oxide (ITO) layer, and the glass, are considered to be the surrounding. Figure 3b shows the longitudinal cross-section of the excitation field without the surrounding, and Figure 3c shows the same field for the case of interaction with the surrounding. The interference can be observed between the forward-propagating and back-reflection excitation field from the substrate. When the excitation field interacts with an 80 nm golden nanosphere standing on the substrate, as shown in Figure 3d, the local field enhancement effect is observed around the nanosphere, which is especially strong at the lower surface. Generally, such a surficial effect on the nanosphere is exponentially decaying in a perpendicular direction to the surface and cannot be detected in the far field; however, the reflection of the substrate may transform this near-field into propagating mode and radiate it into the far field.

Furthermore, the scattered field shown in Figure 3e is obtained by removing the excitation field from the total field. The scattered field decreases with the position of the nanosphere located farther away from the focus. It is worth noting that even though the nanosphere is located very far away, e.g., outside the effective field of view of the objective, a part of the scattered field can be detected since it will be reflected by the substrate, or high order scattering may occur, which may be collected by the objective.

### 2.3. Nanoscale Mapping of the Local Density of State

By considering the light–matter interaction between the nano-object and its surroundings, the final detected field is rather complicated containing reflections of the excitation field from the substrate and the scattered field induced by the nanosphere. Such a phenomenon is closely related to the LDOS of the target. As the target is mainly excited by the z-component of the excitation field and the z-oriented dipoles mainly contribute to the detection field which have been discussed previously, the LDOS of the target can be described by longitudinal component *ρ_z_* as [43,45]:

(9) ρz(rj)=12π2ωIm{Szz(rj,rj)} 

Thus, Equation (8) can be rewritten as:

(10) $$I(r_{j})=\pi^{2}\omega^{2}\alpha^{2}(r_{j})E_{\mathrm{exc},z}^{2}(r_{j})\rho_{z}(r_{j})\;$$

In the linear regime, the transversal component of the total field is much weaker than its longitudinal component that the above radiation can be also expressed as [40]:

(11) $$I(r_{j})=A\alpha^{2}(r_{j})E_{z}^{2}(r_{j})\;$$

In the proposed far-field and non-intrusive CLSM, only the response field of the target in the far-field can be detected. As a result, the signal obtained by the detector through the pinhole for any raster scanning position is derived (see Supplementary Information Section I for detailed derivation) as:

(12) $$I_{\mathrm{im}}(r_{c,j})\propto \sum_{j=1}^{N}PSF_{\det,z}(r_{j})PSF_{exc,z}(r_{j})\alpha^{2}(r_{j})\rho_{z}(r_{j})$$

**Figure 4:**
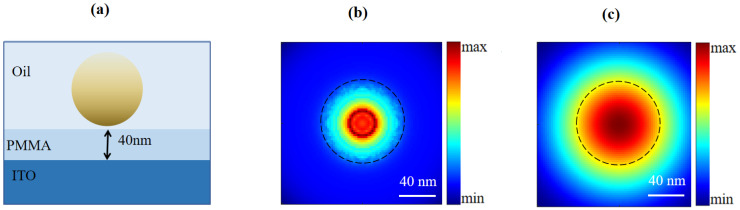
(**a**) The sample of a single nanosphere immersed in oil medium on an ITO glass substrate. (**b**) The corresponding partial LDOS along the longitudinal direction. (**c**) The calculated confocal image.

Equation (12) indicates that the scanning signal of the proposed configuration contains the LDOS of the target, which directly relates to the light mater interaction. However, the resolution is still modulated by the detection of PSF. Therefore, the nanoscale mapping of the nano-objects obtained by the far-field and non-intrusive CLSM is the PSF-modulated LDOS. Figure 4 demonstrates the simulation of the mapping result of an 80 nm nanosphere on a substrate (Figure 4a and Figure S6). Figure 4b is the simulated LDOS of Figure 4a, and Figure 4c shows the simulated scanning image mapped by the proposed CLSM.

## 3. Experimental Results

The far-field non-intrusive CLSM (as shown in Figure 1a) is built to verify the above theory. In the setup, the collimated excitation laser beam from a Nd:YVO_4_ Diode-Pumped Solid State (DPSS) laser (Shenzhen Optoelectronic Technology Co., Ltd., Shenzhen, China), with a wavelength of 405 nm, is converted into an RP beam via a polarization convertor (LPVISE100, Thorlabs, Newton, NJ, USA). The beam then passes through an annular aperture (R1CA2000, Thorlabs, Newton, NJ, USA), and a pair of objectives (Olympus, Tokyo, Japan) are used to match the incident beam with the entrance pupil of the focusing objective; then, the beam enters the back-aperture of a 1.4 NA, 100X oil objective (Olympus, Tokyo, Japan). The sample is placed on the focal plane of the objective and mounted on a high-precision 3D piezo translation stage (P-733.3CD, Physik Instrumente, Karlsruhe, Germany), with a closed-loop accuracy down to 0.3 nm along the x and y directions, and 0.2 nm along the z-axis, to precisely control the sample position during the scanning process. The same objectives are used to collect both reflected and scattered light from the sample in the detection process. After reversely passing through the pairing objectives, the annular aperture, and the polarization convertor, the beam is redirected to a tube lens and eventually focused onto a CCD camera (acA2040-90um, Basler, Ahrensburg, Germany). The collected signals are then processed and reconstructed in a workstation. A single pixel of the CCD, with a pixel size of 5.5 μm, is adopted to work as a pinhole (described in Figure 1b) to block out the out-of-focus field. This configuration is technically possible to be promoted to commercial CLSMs.

**Figure 5:**
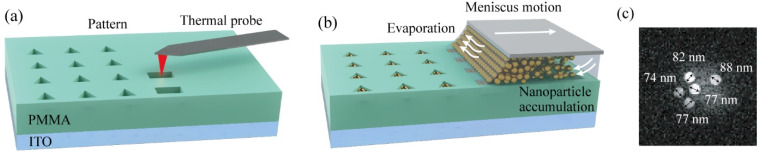
Fabrication of the template and process of capillary-assisted assembly of AuNPs. (**a**) Template fabrication: the designed PMMA template is defined by thermal probe writing. (**b**) Nanoparticle self-assembly: AuNPs accumulate on the pattern via the self-assembly process. (**c**) SEM image of assembled AuNPs on PMMA template, which shows the average lateral dimension of the assembled AuNPs is approximately 80 nm.

**Figure 6:**
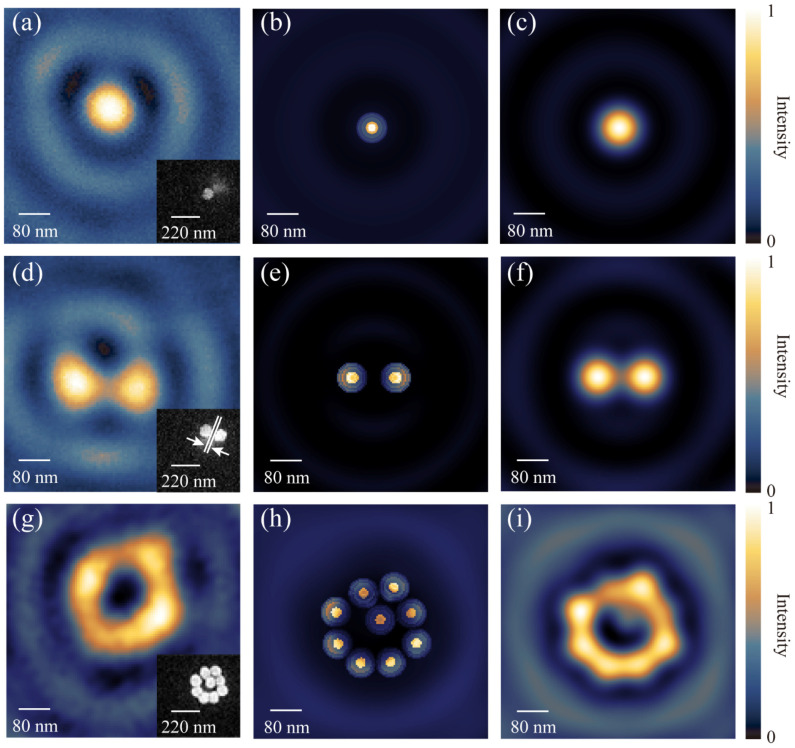
Experiment and simulation results of LDOS mapping. The first column shows the experimentally acquired field distribution by detecting (**a**) a single AuNP, (**d**) two isolated AuNPs, and (**g**) nine nanosphere clusters on the far-field image plane. The second and third columns are the corresponding (**b**,**e**,**h**) simulated LDOS distribution and (**c**,**f**,**i**) PSF-modulated LDOS. Insets on the first column are SEM images of the corresponding samples. Scale bar is 80 nm.

To obtain high-quality nanospheres of the sample, the self-assembled golden nanoparticles (AuNPs) are then fabricated and arranged [46]. The fabrication of the template and the process of AuNPs’ assembly are depicted in Figure 5. The PMMA (Nippon Kayaku, Japan) solution is first spin-coated on a piece of ultrasonically cleaned ITO (JCOPTIX, Nanjing, China) and thereafter dried. The designed patterns formed by a series of 140 nm-deep nanotraps are subsequently transferred onto the 180 nm PMMA layer via thermal probe writing on a NanoFrazor Explore (Heidelberg Instruments Mikrotechnik GmbH, Heidelberg, Germany) platform (Figure 6a) to form the template [47], leaving a 40 nm-thick PMMA layer away from the ITO layer. It is then placed on a stage of a homemade facility to prepare for the assembly process. The self-assembly procedure begins by placing a droplet of colloidal suspensions containing AuNPs with an average size of 80 nm on the topographically structured template. Thereafter, a coverslip is used to cover the droplet, leading to the formation of a meniscus on the front of the coverslip. Thereafter, the template is dragged at an even velocity controlled by an electric motor whilst keeping the coverslip still. Owing to the evaporation, nanoparticles accumulate at the edge of the meniscus moving over the template and are ultimately deposited in the predefined nanotraps on the template (Figure 6b) [48,49,50,51]. The sample is finally dried by nitrogen flow. The quality of the formation of the AuNPs is then characterized by the scanning electronic microscope (SEM, Gemini 500, Carl Zeiss AG, Jena, Germany), as shown in Figure 6c. The SEM image confirms that the average size of the AuNPs is approximately 80 nm. In order to investigate the nanoscale mapping results, various arrangements of AuNPs patterns are also developed.

The samples are then taken into the proposed far-field non-intrusive CLSM for characterization investigation by raster scanning and on-axis detection. The field distribution on the far-field image plane is recorded as shown in Figure 6. Figure 6a shows the scanning mapping of a single AuNP and the inset shows its SEM image. In this case, the LDOS is very close to the direct imaging of the single AuNP with its FWHM at 80 nm, which shows good fidelity to the size obtained by SEM. Figure 6b,c show the simulated LDOS and PSF-modulated LDOS for the nanosphere. These results indicate that the far-field non-intrusive CLSM can perform high-resolution direct imaging for a single nanosphere since its LDOS can be approximated to its morphology. Figure 6d shows the scanning mapping of two AuNPs with a gap of 33 nm measured by SEM (shown as inset), and Figure 6e,f show its simulated LDOS and PSF-modulated LDOS, respectively. Even though the gap is much smaller than the optical resolution, it can still be resolved, since the LDOS contains the information of the light–matter interaction between the target and its surrounding. The interference may enhance the contrast for this configuration by converting the evanescent wave into a propagating wave for resolution enhancement. 

It is worth noting that, in the case of nanosphere clusters, the results contradict the traditional intuition. Figure 6g shows the scanning mapping of a cluster containing nine AuNPs, with eight in a circle and one in the center. It is expected that the central sphere should appear in the optical imaging for sufficient resolution provided by this system. However, the central one is missing in the experimental mapping. Figure 6h simulates the LDOS of these clusters, and that of the central AuNP is found to be much weaker than the peripheral ones (Figure S5); hence, the corresponding information can be hardly interpreted after being modulated by the PSF (shown in Figure 6i). These results indicate that light microscopy records complicate LDOS rather than the purely morphological property of the sample. The target morphology can be mapped only when the far-field mapping of the LDOS coincides with the geometric morphology. Therefore, it presents a necessity to re-examine the correspondence between the mapping and nanoscale structures. This is very important for high-capacity optical storage, where the high-density morphology information may not be retrieved due to the limitation imposed by LDOS to prevent the light from entering the nanoscale entities. The good agreement between the theory and experimental results provides evidence that such a spatial limitation should not be ignored, which also applies to high-capacity optical storage and information retrieval. 

## 4. Conclusions

We have presented a theoretical analysis and experimental study to answer what optical properties of the complex nanostructures are probed by the far-field and non-intrusive super-resolution techniques. By a comprehensive analytical derivation for interaction between the modulated light and the target in a CLSM configuration, it is found that the CLSM does probe the LDOSs in the far field rather than the sample geometric morphology. To verify this point of view, a CLSM with radially polarized (RP) light for illumination is built and the far-field mapping of LDOS is successfully obtained at the optical resolution down to 74 nm. The experiments verify that the target morphology can be correctly mapped only when the far-field mapping of LDOS coincides with the geometric morphology, while light may be blocked from entering the nanostructure medium with weak or missing LDOS, hence invalidating high-density optical information storage and retrieval. We further discuss the characterization for far-field and non-intrusive interaction with nanostructures of different geometric morphology and compare them with those obtainable with the projection of near-field LDOS and scanning electronic microscopic results. We believe the unprecedented findings in this work well describe the intrinsic physics of far-field and non-intrusive techniques to interact with complex photonic and plasmonic nanostructures, and it is also of great significance to guide the design of optical storage and retrieval systems to be more effective by avoiding the blocking of light induced by LDOS.

## Data Availability

Data underlying the results presented in this paper are not publicly available at this time but may be obtained from the authors upon reasonable request.

## Supplementary Materials

The following supporting information can be downloaded at: https://www.mdpi.com/article/10.3390/nano12132274/s1. Figure S1: sketch of the optical vector field modulation (VFM) in excitation subsystem; Figure S2: the calculated focal field around the focal point in oil medium for wavelength of 405 nm; Figure S3: sketch of the optical VFM in detection system for imaging a dipole source located at the focus of a high-NA aplanatic objective lens; Figure S4: (a) and (b) respectively shows amplitude and phase distribution for each element in the detection Dynamic Green tensor; Figure S5: simulated LDOS (second row) and image (third row) distribution for corresponding AuNPs arrangements (first row) with different gap distance between the central AuNP and right adjacent one; Figure S6: the simulated LDOS of a single AuNP shown in Figure 4a in the immersion materials of oil, water, and air, respectively. References [52,53,54,55,56] are cited in the Supplementary Materials.

## References

[1] Lian C., Vagionas C., Alexoudi T., Pleros N., Youngblood N., Ríos C. (2022). Photonic (Computational) Memories: Tunable Nanophotonics for Data Storage and Computing. Nanophotonics.

[2] Meiling J., Mingsi Z., Xiangping L., Yaoyu C. (2019). Research Progress of Super-Resolution Optical Data Storage. Opto-Electron. Eng..

[3] Gu M., Li X., Cao Y. (2014). Optical Storage Arrays: A Perspective for Future Big Data Storage. Light Sci. Appl..

[4] Gu M., Zhang Q., Lamon S. (2016). Nanomaterials for Optical Data Storage. Nat. Rev. Mater..

[5] Tominaga J., Nakano T., Atoda N. (1998). An Approach for Recording and Readout beyond the Diffraction Limit with an Sb Thin Film. Appl. Phys. Lett..

[6] Zhang Q., Xia Z., Cheng Y.-B., Gu M. (2018). High-Capacity Optical Long Data Memory Based on Enhanced Young’s Modulus in Nanoplasmonic Hybrid Glass Composites. Nat. Commun..

[7] Terris B.D., Mamin H.J., Rugar D. (1996). Near-field Optical Data Storage. Appl. Phys. Lett..

[8] Terris B.D., Mamin H.J., Rugar D., Studenmund W.R., Kino G.S. (1994). Near-field Optical Data Storage Using a Solid Immersion Lens. Appl. Phys. Lett..

[9] Tsujioka T., Irie M. (1998). Theoretical Study of the Recording Density Limit of a Near-Field Photochromic Memory. J. Opt. Soc. Am. B.

[10] Partovi A., Peale D., Wuttig M., Murray C.A., Zydzik G., Hopkins L., Baldwin K., Hobson W.S., Wynn J., Lopata J. (1999). High-Power Laser Light Source for near-Field Optics and Its Application to High-Density Optical Data Storage. Appl. Phys. Lett..

[11] Lee W., Zhou Z., Chen X., Qin N., Jiang J., Liu K., Liu M., Tao T.H., Li W. (2020). A Rewritable Optical Storage Medium of Silk Proteins Using Near-Field Nano-Optics. Nat. Nanotechnol..

[12] Parthenopoulos D.A., Rentzepis P.M. (1989). Three-Dimensional Optical Storage Memory. Science.

[13] Cumpston B.H., Ananthavel S.P., Barlow S., Dyer D.L., Ehrlich J.E., Erskine L.L., Heikal A.A., Kuebler S.M., Lee I.-Y.S., McCord-Maughon D. (1999). Two-Photon Polymerization Initiators for Three-Dimensional Optical Data Storage and Microfabrication. Nature.

[14] Hu Y., Zhang Z., Chen Y., Zhang Q., Huang W. (2010). Two-Photon-Induced Polarization-Multiplexed and Multilevel Storage in Photoisomeric Copolymer Film. Opt. Lett..

[15] Grotjohann T., Testa I., Leutenegger M., Bock H., Urban N.T., Lavoie-Cardinal F., Willig K.I., Eggeling C., Jakobs S., Hell S.W. (2011). Diffraction-Unlimited All-Optical Imaging and Writing with a Photochromic GFP. Nature.

[16] Li X., Cao Y., Tian N., Fu L., Gu M. (2015). Multifocal Optical Nanoscopy for Big Data Recording at 30 TB Capacity and Gigabits/Second Data Rate. Optica.

[17] Zhan Q., Leger J. (2002). Focus Shaping Using Cylindrical Vector Beams. Opt. Express.

[18] Dorn R., Quabis S., Leuchs G. (2003). Sharper Focus for a Radially Polarized Light Beam. Phys. Rev. Lett..

[19] Chen W., Zhan Q. (2006). Three-Dimensional Focus Shaping with Cylindrical Vector Beams. Opt. Commun..

[20] Kitamura K., Sakai K., Noda S. (2010). Sub-Wavelength Focal Spot with Long Depth of Focus Generated by Radially Polarized, Narrow-Width Annular Beam. Opt. Express.

[21] Yang L., Xie X., Wang S., Zhou J. (2013). Minimized Spot of Annular Radially Polarized Focusing Beam. Opt. Lett..

[22] Xie X., Chen Y., Yang K., Zhou J. (2014). Harnessing the Point-Spread Function for High-Resolution Far-Field Optical Microscopy. Phys. Rev. Lett..

[23] Yang K., Xie X., Zhou J. (2017). Generalized Vector Wave Theory for Ultrahigh Resolution Confocal Optical Microscopy. J. Opt. Soc. Am. A.

[24] Meng P., Pereira S., Urbach P. (2018). Confocal Microscopy with a Radially Polarized Focused Beam. Opt. Express.

[25] Wang W., Zhang B., Wu B., Li X., Ma J., Sun P., Zheng S., Tan J. (2020). Image Scanning Microscopy with a Long Depth of Focus Generated by an Annular Radially Polarized Beam. Opt. Express.

[26] Kozawa Y., Sakashita R., Uesugi Y., Sato S. (2020). Imaging with a Longitudinal Electric Field in Confocal Laser Scanning Microscopy to Enhance Spatial Resolution. Opt. Express.

[27] Zhang Y., Bai J. (2009). Improving the Recording Ability of a Near-Field Optical Storage System by Higher-Order Radially Polarized Beams. Opt. Express.

[28] Yamanaka Y., Hirose Y., Fujii H., Kubota K. (1990). High Density Recording by Superresolution in an Optical Disk Memory System. Appl. Opt..

[29] Huang C., Bouhelier A., des Francs G.C., Legay G., Weeber J.-C., Dereux A. (2008). Far-Field Imaging of the Electromagnetic Local Density of Optical States. Opt. Lett..

[30] Carminati R., Cazé A., Cao D., Peragut F., Krachmalnicoff V., Pierrat R., De Wilde Y. (2015). Electromagnetic Density of States in Complex Plasmonic Systems. Surf. Sci. Rep..

[31] Asatryan A.A., Busch K., McPhedran R.C., Botten L.C., Martijn de Sterke C., Nicorovici N.A. (2001). Two-Dimensional Green’s Function and Local Density of States in Photonic Crystals Consisting of a Finite Number of Cylinders of Infinite Length. Phys. Rev. E.

[32] Morgenstern M., Klijn J., Meyer C., Getzlaff M., Adelung R., Römer R.A., Rossnagel K., Kipp L., Skibowski M., Wiesendanger R. (2002). Direct Comparison between Potential Landscape and Local Density of States in a Disordered Two-Dimensional Electron System. Phys. Rev. Lett..

[33] Mignuzzi S., Vezzoli S., Horsley S.A.R., Barnes W.L., Maier S.A., Sapienza R. (2019). Nanoscale Design of the Local Density of Optical States. Nano Lett..

[34] Cazé A., Pierrat R., Carminati R. (2013). Spatial Coherence in Complex Photonic and Plasmonic Systems. Phys. Rev. Lett..

[35] Castanié E., Krachmalnicoff V., Cazé A., Pierrat R., De Wilde Y., Carminati R. (2012). Distance Dependence of the Local Density of States in the near Field of a Disordered Plasmonic Film. Opt. Lett..

[36] Imura K., Nagahara T., Okamoto H. (2005). Near-Field Optical Imaging of Plasmon Modes in Gold Nanorods. J. Chem. Phys..

[37] Colas des Francs G., Girard C., Weeber J.-C., Dereux A. (2001). Relationship between Scanning Near-Field Optical Images and Local Density of Photonic States. Chem. Phys. Lett..

[38] Vignolini S., Intonti F., Riboli F., Wiersma D.S., Balet L., Li L.H., Francardi M., Gerardino A., Fiore A., Gurioli M. (2009). Polarization-Sensitive near-Field Investigation of Photonic Crystal Microcavities. Appl. Phys. Lett..

[39] Chicanne C., David T., Quidant R., Weeber J.C., Lacroute Y., Bourillot E., Dereux A., Colas des Francs G., Girard C. (2002). Imaging the Local Density of States of Optical Corrals. Phys. Rev. Lett..

[40] Viarbitskaya S., Teulle A., Marty R., Sharma J., Girard C., Arbouet A., Dujardin E. (2013). Tailoring and Imaging the Plasmonic Local Density of States in Crystalline Nanoprisms. Nat. Mater..

[41] Haberfehlner G., Schmidt F.-P., Schaffernak G., Hörl A., Trügler A., Hohenau A., Hofer F., Krenn J.R., Hohenester U., Kothleitner G. (2017). 3D Imaging of Gap Plasmons in Vertically Coupled Nanoparticles by EELS Tomography. Nano Lett..

[42] Hörl A., Haberfehlner G., Trügler A., Schmidt F.-P., Hohenester U., Kothleitner G. (2017). Tomographic Imaging of the Photonic Environment of Plasmonic Nanoparticles. Nat. Commun..

[43] Girard C., David T., Chicanne C., Mary A., Des Francs G.C., Bourillot E., Weeber J.-C., Dereux A. (2004). Imaging Surface Photonic States with a Circularly Polarized Tip. Europhys. Lett..

[44] Novotny L., Hecht B. (2012). Principles of Nano-Optics.

[45] Girard C., Dujardin E., Baffou G., Quidant R. (2008). Shaping and Manipulation of Light Fields with Bottom-up Plasmonic Structures. New J. Phys..

[46] Meng Y., Cheng G., Man Z., Xu Y., Zhou S., Bian J., Lu Z., Zhang W. (2020). Deterministic Assembly of Single Sub-20 Nm Functional Nanoparticles Using a Thermally Modified Template with a Scanning Nanoprobe. Adv. Mater..

[47] Knoll A.W., Pires D., Coulembier O., Dubois P., Hedrick J.L., Frommer J., Duerig U. (2010). Probe-Based 3-D Nanolithography Using Self-Amplified Depolymerization Polymers. Adv. Mater..

[48] Ni S., Isa L., Wolf H. (2018). Capillary Assembly as a Tool for the Heterogeneous Integration of Micro- and Nanoscale Objects. Soft Matter.

[49] Flauraud V., Mastrangeli M., Bernasconi G.D., Butet J., Alexander D.T.L., Shahrabi E., Martin O.J.F., Brugger J. (2017). Nanoscale Topographical Control of Capillary Assembly of Nanoparticles. Nat. Nanotechnol..

[50] Kraus T., Malaquin L., Schmid H., Riess W., Spencer N.D., Wolf H. (2007). Nanoparticle Printing with Single-Particle Resolution. Nat. Nanotechnol..

[51] Ni S., Leemann J., Wolf H., Isa L. (2015). Insights into Mechanisms of Capillary Assembly. Faraday Discuss..

[52] Liu Z., Agarwal K. (2020). Silicon Substrate Significantly Alters Dipole-Dipole Resolution in Coherent Microscope. Opt. Express.

[53] Leutenegger M., Rao R., Leitgeb R.A., Lasser T. (2006). Fast Focus Field Calculations. Opt. Express.

[54] Lin J., Rodríguez-Herrera O.G., Kenny F., Lara D., Dainty J.C. (2012). Fast Vectorial Calculation of the Volumetric Focused Field Distribution by Using a Three-Dimensional Fourier Transform. Opt. Express.

[55] Yurkin M.A., Hoekstra A.G. (2007). The Discrete Dipole Approximation: An Overview and Recent Developments. J. Quant. Spectrosc. Radiat. Transfer.

[56] Schmehl R., Nebeker B.M., Hirleman E.D. (1997). Discrete-Dipole Approximation for Scattering by Features on Surfaces by Means of a Two-Dimensional Fast Fourier Transform Technique. J. Opt. Soc. Am. A.

