# Supplementary material for: Far-Field and Non-Intrusive Optical Mapping of Nanoscale Structures
Source: Nanomaterials (Basel). 2022 Jul 1;12(13):2274. doi: 10.3390/nano12132274 (PMC9268055; doi:10.3390/nano12132274)
Supplement: Supplementary file 1 [file nanomaterials-12-02274-s001.zip › nanomaterials-1795086-supplementary.pdf]

nanomaterials

Supplementary Materials

Far-Field and Non-Intrusive Optical Mapping of Nanoscale Structures

Guorong Guan [1,†], Aiqin Zhang [1,†], Xiangsheng Xie [2], Yan Meng [3], Weihua Zhang [3], Jianying Zhou [1,*] and Haowen Liang [1,*]

[1] State Key Laboratory of Optoelectronic Materials and Technologies, School of Physics, School of Electronics and Information Technology, Sun Yat-Sen University, Guangzhou 510275, China; guangr@mail2.sysu.edu.cn (G.G.); aiqin_zhang@126.com (A.Z.)
[2] Department of Physics, College of Science, Shantou University, Shantou 515063, China; xxs@stu.edu.cn
[3] State Key Laboratory of Analytical Chemistry for Life Science, MOE Key Laboratory of Intelligent Optical Sensing and Manipulation, Jiangsu Key Laboratory of Artificial Functional Materials, College of Engineering and Applied Sciences, Nanjing University, Nanjing 210093, China; skywalker16@126.com (Y.M.); zwh@nju.edu.cn (W.Z.)
* Correspondence: stszjy@mail.sysu.edu.cn (J.Z.); lianghw26@mail.sysu.edu.cn (H.L.)
† These authors contributed equally to this work.

I. Theory

1. Excitation Field

Benefiting from the sub-wavelength focal spot with long depth of focus [20–23], the focusing field of a high numerical aperture annular RP beam can be utilized as a virtual optical nanoneedle to probe the sample in a CLSM. As shown in Figure S1, RP beam (denoted as $\mathbf{E}_{in}(\rho)$)in the excitation process is generated by applying a polarization converter (PC) to the linear polarization (LP) laser beam propagating along the optical axis, originating from a point source. Subsequently, it is filtered by an annular aperture so that only the light carrying high spatial frequencies can pass. In our setup, the passing light carries the spatial frequency corresponding closely to the maximum one that an oil immersed objective lens with NA of 1.4 allows to collect.

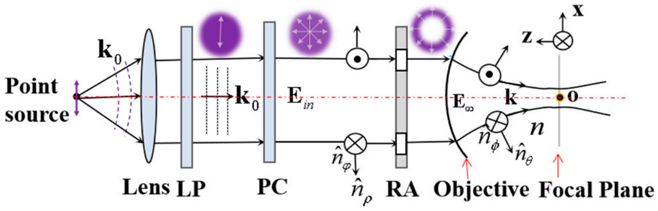

Figure S1. Sketch of the optical vector field modulation (VFM) in excitation subsystem. $\mathbf{k}_0 = 2\pi c/\lambda$ and $\mathbf{k} = n\mathbf{k}_0 = (k_x, k_y, k_z)$ is respectively the wave vector in free space with refractive index of $n_0 = 1$ and in the oil immersion medium with refractive index of n = 1.515. And the intersection point O of the focal plane and optical axis (red dot line) is set as the focal point of the objective lens, and also the original point of the cartesian coordinate system $(\hat{n}_x \;\; \hat{n}_y \;\; \hat{n}_z)$ .The unit vector $\hat{n}_\rho$, $\hat{n}_\varphi$ in the cylindrical coordinate systems and $\hat{n}_\theta$, $\hat{n}_\phi$ in spherical coordinate systems can be both expressed in terms of the Cartesian unit vectors $\hat{n}_x$, $\hat{n}_x$, $\hat{n}_x$ using the spherical coordinates θ and ϕ , as $\hat{n}_\rho = \cos\phi \hat{n}_x + \sin\phi \hat{n}_y$; $\hat{n}_\phi = -\sin\phi \hat{n}_x + \cos\phi \hat{n}_y$; $\hat{n}_\theta = \cos\theta\cos\phi \hat{n}_x + \cos\theta \sin\phi \hat{n}_y - \sin\theta \hat{n}_z$.

The focusing field $\mathbf{E}_f(\mathbf{r})$ at any point $\mathbf{r}(x,y,z)$ in object plane can be obtained from its corresponding angular spectrum $\hat{\mathbf{E}}_f(k_x,k_y;z)$ by the inverse Fourier transform (iFT)

$$\mathbf{E}_f(x,y,z)=\iint_{k_x,k_y}\hat{\mathbf{E}}_f(k_x,k_y;z)e^{i[k_x x+k_y y]}dk_x dk_y \quad \text{(S1)}$$

And the angular spectrum $\hat{\mathbf{E}}_f(k_x,k_y;z)$ evolves along the z axis can be obtained from that on the focal plane $\hat{\mathbf{E}}_f(k_x,k_y;0)$ by multiplying the propagating factor $e^{ik_z z}$ as

$$\hat{\mathbf{E}}_f(k_x,k_y;z)=\hat{\mathbf{E}}_f(k_x,k_y;0)\cdot e^{ik_z z} \quad \text{(S2)}$$

According to the method of stationary phase [44,52], the angular spectrum at the focal plane can be related to its far-field $\hat{\mathbf{E}}_\infty(k_x,k_y)$ by:

$$\hat{\mathbf{E}}_f(k_x,k_y;0)=\frac{ife^{-ikf}}{2\pi ik_z}\mathbf{E}_\infty(k_x,k_y) \quad \text{(S3)}$$

Here the far-field can be regarded as the transmitted field immediately after the objective lens, f is the equivalent focal length of the objective lens. The aplanatic objective is described by two rules[5]: the sine condition $\rho=f\sin(\theta)$ and the intensity conservation law. Thus, the far-field can be derived from the following expression

$$\mathbf{E}_\infty(k_x,k_y)=\mathbf{E}_\infty(\theta,\phi)=\left[(\mathbf{E}_{in}\cdot\hat{n}_\rho)\hat{n}_\theta+(\mathbf{E}_{in}\cdot\hat{n}_\varphi)\hat{n}_\phi\right]P(\theta,\varphi)\sqrt{\frac{\cos\theta}{n}} \quad \text{(S4)}$$

The RP incident field $\mathbf{E}_{in}$ before entering into the annular aperture can be expressed as:

$$\mathbf{E}_{in}=\mathrm{E}_0(\rho)\hat{\mathrm{n}}_\rho \quad \text{(S5)}$$

Generally, the amplitude $\mathrm{E}_0(\rho)$ for the RP beam has a singularity in the center of the optical spot. According to the sine condition at the objective lens and coordinate transform[5], it can also be expressed as $\mathrm{E}_0(\rho)=\mathrm{E}_0(k_x,k_y)$. $P(\theta,\phi)$ in Eq. (1.4) denotes the apodization function determined by the parameters of the annular aperture and the objective lens, with the expression:

$$P(\theta,\phi)=P(k_x,k_x)=\begin{cases}1 & (\mathcal{R}NA\cdot k_0\le\sqrt{k_x^2+k_y^2}\le NA\cdot k_0)\\ 0 & (\sqrt{k_x^2+k_y^2}\le\mathcal{R}NA\cdot k_0 \quad or \quad \sqrt{k_x^2+k_y^2}\ge NA\cdot k_0)\end{cases} \quad \text{(S6)}$$

$\mathcal{R}=r_1/r_2$, r_1 and r_2 are the radii of the inner and outer of the annular aperture, respectively, and r_2 should be set equal to that of the back aperture of the objective lens (denoted as r_0). In experiment, in order to reduce the loss of energy, an annular aperture with ratio $\mathcal{R}=0.85$ and $r_2=0.5r_0$ is utilized, and subsequently a 2× magnification to the annular beam is applied, realized by an objective lens pair.

Combining the Equations S1–S6, the transform formulas in the right side of the optical path map as shown in Figure 1 can be denoted as $\mathbf{E}_{exc}(\mathbf{r})$ and is derived to have the following expression

$$\mathbf{E}_{exc}(\mathbf{r})=\begin{bmatrix}E_{exc,x}\\E_{exc,y}\\E_{exc,z}\end{bmatrix}=\frac{ife^{-ikf}}{2\pi}\mathcal{F}^{-1}\left\{\frac{E_0(k_x,k_y)P(k_x,k_y)}{k\sqrt{(k_x^2+k_y^2)}}\sqrt{\frac{k_z}{nk}}\begin{bmatrix}k_xk_z\\k_yk_z\\-(k_x^2+k_y^2)\end{bmatrix}\frac{e^{ik_zz}}{k_z}\right\} \tag{S7}$$

This focusing field of the incident RP annular beam then is used as the excitation field in a CLSM. The corresponding PSF for a point source manipulated by excitation process as shown in Fig.S1 is

$$\mathbf{PSF}_{exc}=\mathbf{E}_{exc}\cdot\mathbf{E}^*_{exc}=\begin{bmatrix}PSF_{exc,x}\\PSF_{exc,y}\\PSF_{exc,z}\end{bmatrix}=\begin{bmatrix}E_{exc,x}\cdot E^*_{exc,x}\\E_{exc,y}\cdot E^*_{exc,y}\\E_{exc,z}\cdot E^*_{exc,z}\end{bmatrix} \tag{S8}$$

Therefore, the transversal component of the PSF is: $PSF_{exc,//}=PSF_{exc,x}+PSF_{exc,y}$.

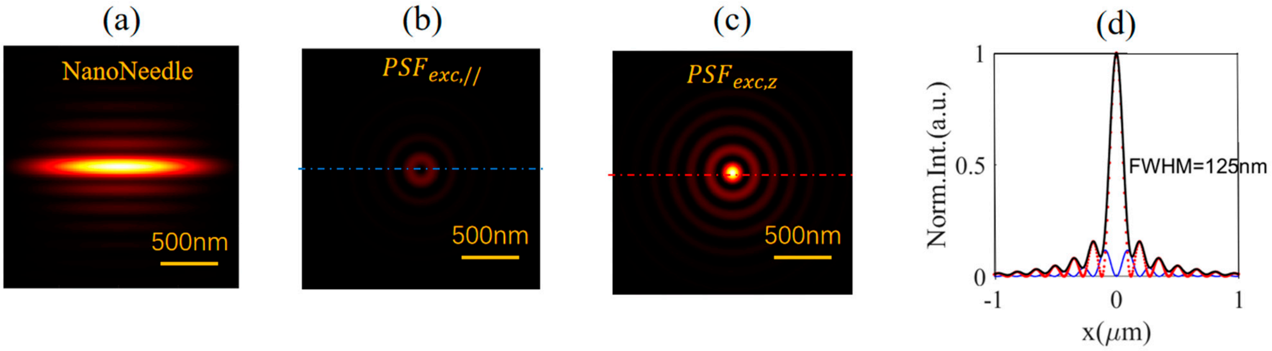

Figure S2. The calculated focal field around the focal point in oil medium for wavelength of 405 nm. (a) the intensity distribution of the focusing nanoneedle in the through focus XZ plane. The transversal (b) and longitudinal (c) polarization component of the excitation PSF in the focal plane. (d) The intersection of the intensity profile for the total (black) excitation PSF and its transversal (blue) and longitudinal (red) components in the focal plane.

By using chirp z transform (CZT) [53,54], the focusing field can be quickly calculated. From the calculated results as demonstrated in Figure S2a, it is clear that the incident RP annular beam has evolved into an optical nanoneedle around the focal point due to the tightly focusing by the objective lens. The central intensity of the transversal component in Figure S2b is much smaller compared with that of the longitudinal component in Figure S2c. Therefore, the linear excitation of the focal field to matter can be approximately regarded as induced by the longitudinal polarization component. Figure S2d shows the intersection of the focusing profile, which gives a more intuitive contrast between the transversal and longitudinal components for the excitation PSF. The FWHM of the total excitation PSF is about 125nm.

2. Detection Point Spread Function

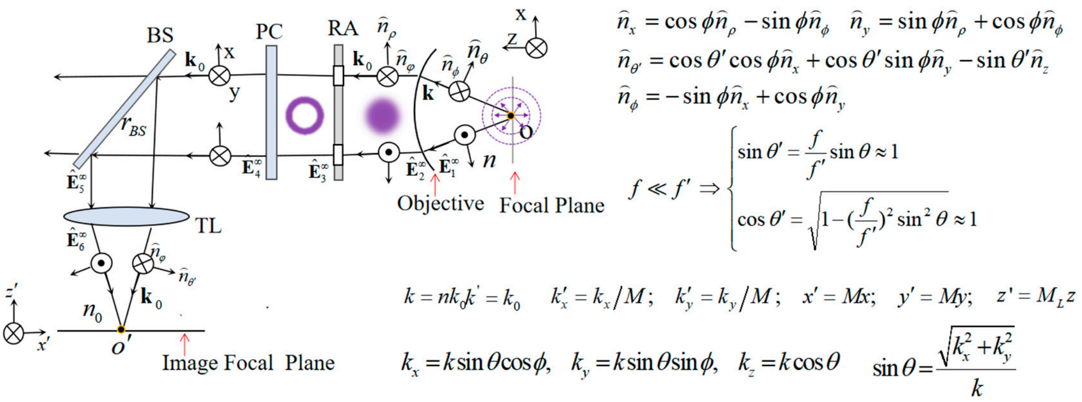

Figure S3. Sketch of the optical VFM in detection system for imaging a dipole source located at the focus of a high-NA aplanatic objective lens. The surrounding space of the dipole is assumed to be a homogeneous medium with refractive index of n = 1.515. O' in image space is the conjugate point of focal point O in object space. And they are respectively the origin for the Cartesian coordinate system in object and image space.

As demonstrated in Figure S3, the radiating field from an induced dipole is firstly collected by the same objective lens. During this process, the optical field element along the $\hat{n}_\theta$ and $\hat{n}_\phi$ in sphere coordinate in object space is respectively transformed into the RP and AP elements along $\hat{n}_\rho$ and $\hat{n}_\varphi$ in cylindrical coordinate systems. With the opposite direction passing through PC, the RP and AP beam elements of the collected dipole field after being filtered by the annular aperture, is subsequently transformed into two orthogonal polarized light along $\hat{n}_x$ and $\hat{n}_y$ in Cartesian coordinate systems. Reflected by the beam splitter (BS), it will be focused by a tube lens which matches the used oil objective lens. In the following content in this part, the mathematical derivation process will be demonstrated for the modulation of the detection PSF.

The electric field $\mathbf{E}_1(\mathbf{r})$ at any point $\mathbf{r}(x,y,z)$ in the object space, that emitting from a free space point source $\boldsymbol{\mu}_0$ located at focus $\mathbf{r}_0(0,0,0)$, can be defined by the free-space dyadic Green function $\overleftrightarrow{\mathbf{G}}_0(\mathbf{r},\mathbf{r}_0)$ according to $\mathbf{E}_1(\mathbf{r}) = \frac{\omega^2}{\varepsilon_0 c^2}\overleftrightarrow{\mathbf{G}}_0(\mathbf{r},\mathbf{r}_0)\cdot\boldsymbol{\mu}_0$. In angular spectrum representation, it can also be rewritten as

$$\mathbf{E}_1(\mathbf{r}) = \iint \hat{\mathbf{E}}_0(k_x,k_y;0)e^{ik_z z}e^{i[k_x x + k_y y]}dk_x dk_y \cdot \boldsymbol{\mu}_0 \tag{S9}$$

Here the angular spectrum at the focal plane has the following expression[5]

$$\hat{\mathbf{E}}_0(k_x,k_y;0) = \frac{\omega^2}{\varepsilon_0 c^2}\cdot\frac{i}{8\pi^2}\{[\mathbf{M}^S + \mathbf{M}^P\} \tag{S10}$$

$\mathbf{M}^S$, $\mathbf{M}^P$ respectively denotes the angular spectrum for s- and p- polarization element of the free space Green function $\overleftrightarrow{\mathbf{G}}_0$.

According to the method of stationary phase, the far field of the free space dipole radiating in sphere coordinate system $(\hat{n}_r\ \hat{n}_\theta\ \hat{n}_\phi)$ can be obtained by the following relationship,

$$\hat{\mathbf{E}}_1^\infty(\theta,\phi) = -2\pi i k_z \hat{\mathbf{E}}_0(k_x,k_y,0)\frac{e^{ikr}}{r}\cdot\boldsymbol{\mu}_0 \tag{S11}$$

After passing through the objective lens, the transmitted field in cylindrical coordinates $(\hat{n}_\rho\ \hat{n}_\varphi\ \hat{n}_z)$ is expressed as

$$\hat{\mathbf{E}}_2^{\infty}(\theta,\varphi)=\left\{\left[\hat{\mathbf{E}}_1^{\infty}(\theta,\phi)\cdot\hat{n}_\phi\right]\hat{n}_\phi+\left[\hat{\mathbf{E}}_1^{\infty}(\theta,\phi)\cdot\hat{n}_\theta\right]\hat{n}_\rho\right\}\sqrt{\frac{n}{\cos\theta}} \quad \text{(S12)}$$

Subsequently, it is filtered by the annular aperture as

$$\hat{\mathbf{E}}_3^{\infty}(\theta,\varphi)=\mathbf{E}_2^{\infty}(\theta,\varphi)\cdot P(\theta,\varphi) \quad \text{(S13)}$$

Then, a polarization conversion is applied to the annular beam, making the RP element and AP element transform into two orthogonal linear polarized states, as expressed in a Cartesian coordinate system

$$\hat{\mathbf{E}}_4^{\infty}(k_x,k_y)=\left\{\left[\hat{\mathbf{E}}_3^{\infty}(k_x,k_y)\cdot\hat{n}_\rho\right]\hat{n}_x+\left[\hat{\mathbf{E}}_3^{\infty}(k_x,k_y)\cdot\hat{n}_\phi\right]\hat{n}_y\right\} \quad \text{(S14)}$$

By expressing the orthogonal LP light in the cylindrical coordinates, and assuming the reflection coefficient of beam splitter to S-polarization is equal to that of the P-polarization beam, the field at the back aperture of the tube lens can be described by:

$$\hat{\mathbf{E}}_5^{\infty}(k_x,k_y)=r_{BS}\left\{\left[\mathbf{E}_4^{\infty}(k_x,k_y)\cdot\hat{n}_x\right](\cos\phi\hat{n}_\rho-\sin\phi\hat{n}_\phi)+\left[\mathbf{E}_{2'}^{\infty}(k_x,k_y)\cdot\hat{n}_y\right](\sin\phi\hat{n}_\rho+\cos\phi\hat{n}_\phi)\right\} \quad \text{(S15)}$$

The field immediately after the tube lens is:

$$\hat{\mathbf{E}}_6^{\infty}(k'_x,k'_y)=\left\{\left[\hat{\mathbf{E}}_5^{\infty}(k_x,k_y)\cdot\hat{n}_\rho\right]\hat{n}_{\theta'}+\left[\hat{\mathbf{E}}_5^{\infty}(k_x,k_y)\cdot\hat{n}_\varphi\right]\hat{n}_\phi\right\}\sqrt{\cos\theta'} \quad \text{(S16)}$$

And the angular spectrum on the image focal plane is:

$$\hat{\mathbf{E}}_0(k_{x'},k_{y'};0)=\frac{if'e^{-ik_0f'}}{2\pi ik_{z'}}\cdot\hat{\mathbf{E}}_6^{\infty}(k'_x,k'_y) \quad \text{(S17)}$$

By using the iFT, the field at any point $\mathbf{r}'(x',y',z')$ in image space is:

$$\mathbf{E}_1(\mathbf{r}')=\iint\hat{\mathbf{E}}_0(k_{x'},k_{y'};0)e^{i[k'_xx'+k'_yy'+k'_zz']}dk'_xdk'_y \quad \text{(S18)}$$

As the relationship between the dipole in object and its corresponding field in image space can be obtained by the process $\mathbf{E}_1(x',y',z')=\frac{\omega^2}{\varepsilon_0c^2}\overleftrightarrow{\mathbf{G}}_{\mathrm{det}}(x',y',z')\boldsymbol{\mu}_0$, the Green dynamic function for the free space dipole radiating after being collected and modulated by the detection system is derived to have the following expression:

$$\overleftrightarrow{\mathbf{G}}_{det}(x',y',z')=\overleftrightarrow{\mathbf{G}}_{det}(xM,yM,zM^2/n)=A_{det}^0\cdot\mathcal{F}^{-1}\left\{\mathbf{T}_{\mathrm{det}}(k_x,k_y,k_z)\right\} \quad \text{(S19)}$$

where $A_{\mathrm{det}}^0=\frac{f'e^{i(kf-k_0f')}}{8\pi^2fk_0M^2}$, f' is effective focal length for the tube lens, M is the magnification of the detection system. And its angular spectrum for the field in image space is described as:

$$\mathbf{T}_{det}(k_x,k_y,k_z)=\mathcal{A}_{det}\left[\mathbf{T}^x(k_x,k_y,k_z)\quad\mathbf{T}^y(k_x,k_y,k_z)\quad\mathbf{T}^z(k_x,k_y,k_z)\right] \quad \text{(S20)}$$

where $\mathcal{A}_{det}=\frac{P(k_x,k_y)k_z}{(k_x^2+k_y^2)^{3/2}}\sqrt{\frac{nk}{k_z}}e^{ikz\left[\left(\frac{f'}{f}\right)^2-\frac{1}{2}\frac{k_x^2+k_y^2}{k^2}\right]}e^{-ik_zz_0}$, z_0 denotes the distance between the focal plane and the upper surface of a multilayer planar substrate structure. $\mathbf{T}^x$, $\mathbf{T}^y$

and $\mathbf{T}^z$ respectively denotes the detected spatial frequency for the dipole that along the basic coordinate axis of Cartesian coordinate system. And each element has the expression:

$$\mathbf{T}^x = \begin{bmatrix} (k_x^3 k_z^2 + k_x k_y^2 k_z^2 + k_x (k_x^2 + k_y^2)^2)/k^3 \\ (-k_y^3 - k_x^2 k_y)/k_z \\ 0 \end{bmatrix} \tag{S21}$$

$$\mathbf{T}^y = \begin{bmatrix} (k_y^3 k_z^2 + k_x^2 k_y k_z^2 + k_y (k_x^2 + k_y^2)^2)/k^3 \\ (k_x^3 + k_x k_y^2)/k_z \\ 0 \end{bmatrix} \tag{S22}$$

$$\mathbf{T}^z = \begin{bmatrix} [-k_x^2 k_z (k_x^2 + k_y^2) - k_y^2 k_z (k_x^2 + k_y^2) - (k_x^2 + k_y^2)^3 / k_z]/k^3 \\ 0 \\ 0 \end{bmatrix} \tag{S23}$$

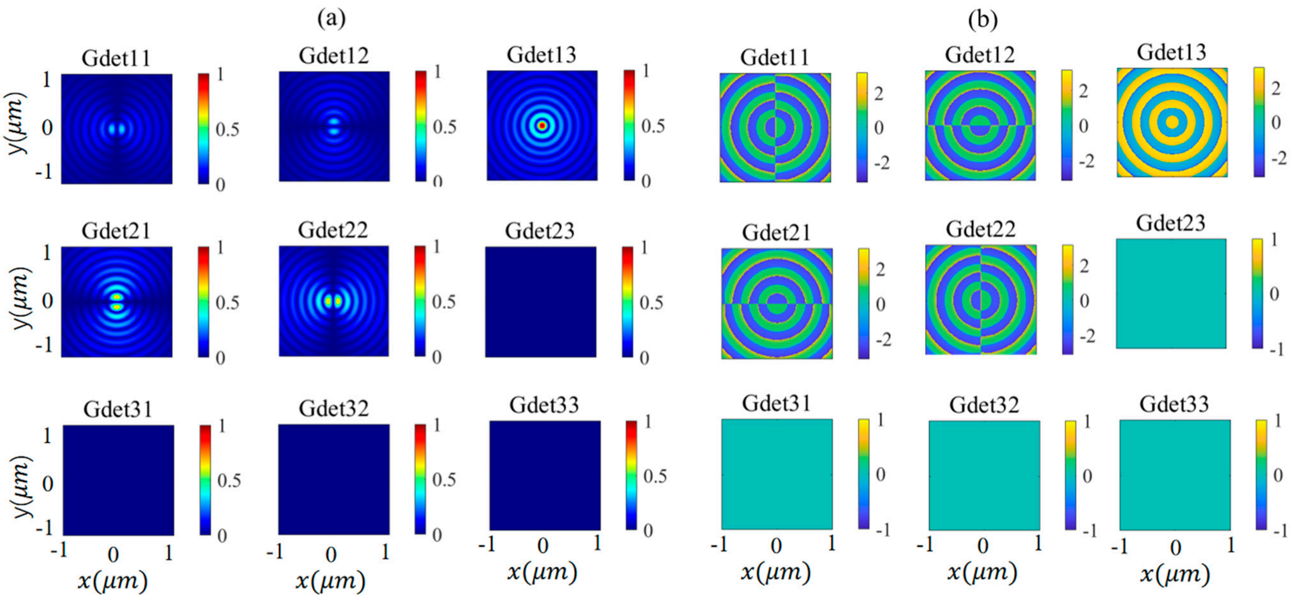

Figure S4. (a) and (b) respectively shows amplitude and phase distribution for each element in the detection Dynamic Green tensor. It can be seen that the detected Green function is dark at center for dipole oscillating along x- and y- direction. Thus, if a small pinhole is applied, the detected signal is mainly originating from radiating of the z-oriented dipole.

And the PSF for a dipole after being detected and manipulation by the detection system can be expressed as:

$$\mathbf{PSF}_{det}=\begin{bmatrix}PSF_{det,x}\\PSF_{det,y}\\PSF_{det,z}\end{bmatrix}=\begin{bmatrix}\sum_{i_x=1}^{3}\overleftrightarrow{\mathbf{G}}_{\det}\{i_x,1\}\cdot\overleftrightarrow{\mathbf{G}}^{*}_{\det}\{i_x,1\}\\\sum_{i_y=1}^{3}\overleftrightarrow{\mathbf{G}}_{\det}\{i_y,2\}\cdot\overleftrightarrow{\mathbf{G}}^{*}_{\det}\{i_y,2\}\\\sum_{i_z=1}^{3}\overleftrightarrow{\mathbf{G}}_{\det}\{i_z,3\}\cdot\overleftrightarrow{\mathbf{G}}^{*}_{\det}\{i_z,3\}\end{bmatrix} \quad \text{(S24)}$$

3. Scanning Image and Light Matter Interaction

In experiment, the signal detected by the co-axial pixels of a CCD with the pixel size of 5 μm is used to reconstruct the scanning image for a sample. To the far-field working mode of the proposed supper-resolution CLSM, the spatial linear translation invariance is theoretically effective, for obtaining the response field from arbitrary local position inside the sample that scanning near the focal point in object plane, i.e., the field of a dipole induced by the lateral offset focal point will also be collected by the objective and therefore influences the imaging. Thus, the image field filtered by the equivalent pinhole area for a dipole moment $\mathbf{P}(\mathbf{r})$ at $\mathbf{r}(x,y,z)$ in object plane can be expressed as $\frac{\omega^2}{\varepsilon_0 c^2}\overleftrightarrow{\mathbf{G}}_{\det}(xM,yM,zM^2/n)\cdot\mathbf{P}(\mathbf{r})$. With a discrete dipole approximation (DDA) model for the sample, the total optical electric field projecting on the co-axial pixel should be the summation of the responding field from all discrete dipoles inside the sample, as:

$$\mathbf{E}_{im}(\mathbf{r}_{c,i})=\frac{\omega^2}{\varepsilon_0 c^2}\sum_{j=1}^{N}\overleftrightarrow{\mathbf{G}}_{\det}(x_jM,y_jM,z_jM^2/n)\cdot\mathbf{P}(\mathbf{r}_j) \quad \text{(S25)}$$

Here $\mathbf{r}_{c,i}$ denotes the relative position of the sample to the optical axis of the CLSM at the ith scanning, N is the number of the induced dipoles inside the sample, $\mathbf{r}_j$ denotes the position of an approximate dipole for an elementary cell with volume of v_j inside the sample.

As only the field of z-oriented dipole is nonzero at the center, thus the detection Green function can be approximated to:

$$\overleftrightarrow{\mathbf{G}}_{\det}\approx\begin{bmatrix}0&0&G_{det,zx}\\0&0&0\\0&0&0\end{bmatrix} \quad \text{(S26)}$$

where $G_{det,zx}$ is the x-component of the detected field for z-oriented dipole.

And the detected signal used to register as the pixel information of the reconstructed scanning image is directly proportional to the projecting optical intensity on the co-axial pixel as:

$$I_{im}(\mathbf{r}_{c,j})=\mathbf{E}_{im}(\mathbf{r}_{c,j})\cdot\mathbf{E}^{*}_{im}(\mathbf{r}_{c,j}) \quad \text{(S27)}$$

In linear response theory, when the excitation nano-field generated by focusing the RP beam is applied to the sample, the induced dipole moment $\mathbf{P}(\mathbf{r}_j)$ for an elementary cell can be related to the total field $\mathbf{E}(\mathbf{r}_j)$ by the dipole polarizability $\boldsymbol{\alpha}(\mathbf{r}_j)$ through [55]:

$$\mathbf{P}(\mathbf{r}_j) = \boldsymbol{\alpha}(\mathbf{r}_j) \cdot \mathbf{E}(\mathbf{r}_j) \tag{S28}$$

Generally, the Clausius-Mossotti polarizabilities $\alpha_j^{CM} = \frac{3d^3}{4\pi}\frac{\varepsilon_j - 1}{\varepsilon_j + 2}$ can be used, where ε_j is the dielectric function at dipole position $\mathbf{r}_j$,and d is spacing between dipoles.

The actual excitation field $\mathbf{E}(\mathbf{r}_j)$ inside a nano-particle for the case that put on a substrate can be regarded as the summation of incident field, which includes the direct excitation field and its reflected part from the surface of the substrate; the field created by direct interaction between the dipoles; and the dipole field reflected from the surface [56].

With the introduction of so-called field susceptibility $\overleftrightarrow{\mathbf{S}}(\mathbf{r},\mathbf{r}_j)$ 11, the field $\mathbf{E}(\mathbf{r})$ generated by a dipole in a practical photonic environment can be related to the initial dipole moment $\mathbf{P}_0(\mathbf{r}_j)$ induced directly by the RP focusing field.

$$\mathbf{E}(\mathbf{r}) = \overleftrightarrow{\mathbf{S}}(\mathbf{r},\mathbf{r}_j) \cdot \mathbf{P}_0(\mathbf{r}_j) \tag{S29}$$

And $\overleftrightarrow{\mathbf{S}}(r,r_j)$ can be also called as the total Green function for the dipole $\mathbf{P}_0(\mathbf{r}_j)$, which including the free space Green function $\overleftrightarrow{\mathbf{G}}_0(\mathbf{r},\mathbf{r}_j)$ and its scattered parts $\overleftrightarrow{\mathbf{G}}_s(\mathbf{r},\mathbf{r}_j)$ from its surroundings as $\overleftrightarrow{\mathbf{S}}(\mathbf{r},\mathbf{r}_j) = \overleftrightarrow{\mathbf{G}}_0(\mathbf{r},\mathbf{r}_j) + \overleftrightarrow{\mathbf{G}}_s(\mathbf{r},\mathbf{r}_j)$.

Without considering the weak RP element of the incident focusing field, the initially induced dipole inside the sample can be denoted as

$$\mathbf{P}_0(\mathbf{r}_j) = [0, \quad 0, \quad \boldsymbol{\alpha}(\mathbf{r}_j)\mathbf{E}_{exc,z}(\mathbf{r}_j)]^T \tag{S30}$$

Combining Eq. (3.1) to (3.6), the pixel information for the scanning image is derived to have the following expression

$$I_{im}(\mathbf{r}_{c,j}) = \mu_0^2 \omega^4 \sum_{j=1}^{N} PSF_{det,z}(\mathbf{r}_j)\alpha^2(\mathbf{r}_j)E_z^2(\mathbf{r}_j) \tag{S31}$$

where $E_z(\mathbf{r}_j)$ denotes the z-component of total field $\mathbf{E}(\mathbf{r}_j)$

4. Imaging of the Local Density of State

The radiation energy from a dipole at $\mathbf{r}_j$ can be obtained by

$$I(\mathbf{r}_j) = -\frac{1}{2}\int_V d\mathbf{r}\, Re\left[\mathbf{J}(\mathbf{r}) \cdot \mathbf{E}(\mathbf{r})\right] \tag{S32}$$

Here, the current density for an induced dipole located at $\mathbf{r}_j$ is expressed as

$$\mathbf{J}(\mathbf{r}) = -i\omega\mathbf{P}_0(\mathbf{r}_j)\delta(\mathbf{r}-\mathbf{r}_j) \tag{S33}$$

Combining the Equations S29, S32 and S33,

$$I(\mathbf{r}_j) = \frac{\omega}{2}\left[\boldsymbol{\alpha}(\mathbf{r}_j)\mathbf{E}_{exc,z}(\mathbf{r}_j)\right]^2 Im\left\{S_{zz}(\mathbf{r}_j,\mathbf{r}_j)\right\} \tag{S34}$$

And by utilizing the relationship between the partial local density of state (LDOS) and total Green dynamic function tensor [43]

$$\rho_z(\mathbf{r}_j) = \frac{1}{2\pi^2\omega} Im\left\{S_{zz}(\mathbf{r}_j,\mathbf{r}_j)\right\} \tag{S35}$$

The Eq. (4.3) can be rewritten as

$$I(\mathbf{r}_j) = \pi^2\omega^2\boldsymbol{\alpha}^2(\mathbf{r}_j)\mathbf{E}^2_{exc,z}(\mathbf{r}_j)\rho_z(\mathbf{r}_j) \tag{S36}$$

In general [40], if the weak transversal component of the actual total field $\mathbf{E}(\mathbf{r})$ is ignored, the energy radiated by elementary cell inside the scattering system in a linear process can also be expressed as

$$I(\mathbf{r}_j) = A\boldsymbol{\alpha}^2(\mathbf{r}_j)\mathbf{E}_z^2(\mathbf{r}_j) \tag{S37}$$

Thus, we can get that

$$\mathbf{E}_z{}^2(\mathbf{r}_j) = A^{-1}\pi^2\omega^2\mathbf{E}^2_{exc,z}(\mathbf{r}_j)\rho_z(\mathbf{r}_j) \tag{S38}$$

Inserting the expression of $\mathbf{E}_z{}^2(\mathbf{r}_j)$ in Equation S31, the pixel information for scanning image can be expressed as

$$I_{im}(\mathbf{r}_{c,j}) \propto \sum_{j=1}^{N} PSF_{det,z}(\mathbf{r}_j)PSF_{exc,z}(\mathbf{r}_j)\alpha^2(\mathbf{r}_j)\rho_z(\mathbf{r}_j) \tag{S39}$$

Eq. (4.8) tells that the scanning signal of the proposed configuration contains the LDOS of the target, which directly describes any light mater interaction results of the target; yet the resolution is still modulated by the detected PSF.

II. FDTD simulation condition

In the FDTD simulation, the complex refractive index of Au is obtained using data from Johnson and Christy in the materials database. The refractive indices of oil, PMMA, glass, and ITO are set to 1.515, 1.5, 1.45, and 2.0574 + 0.0113i, respectively. A self-defined source according to Equation 1 is set at the distance of z = 1.5 μm above the substrate as the excitation field, where the ITO upper surface is set as z = 0. The mesh with the precision of 2 nm is applied near the NPs areas within a 2 μm volume, while the auto non-uniform mesh type is used for reducing the simulation time out of this volume. And the perfect matched layers are used for all the boundary conditions.

III. The LDOS of AuNP Cluster with Different Gap Distance between the Central One and Peripheral Ones

We simulate the LDOS of nine-sphere cluster with different gap distances from 0 nm to 20 nm between the central AuNP and the adjacent one. The increment is 5 nm. It is clearly seen that the intensity of LDOS of the central AuNP keeps very weak in all cases. Therefore, the central AuNP cannot be resolved no matter what the gap distances are, as shown in Figure S5.

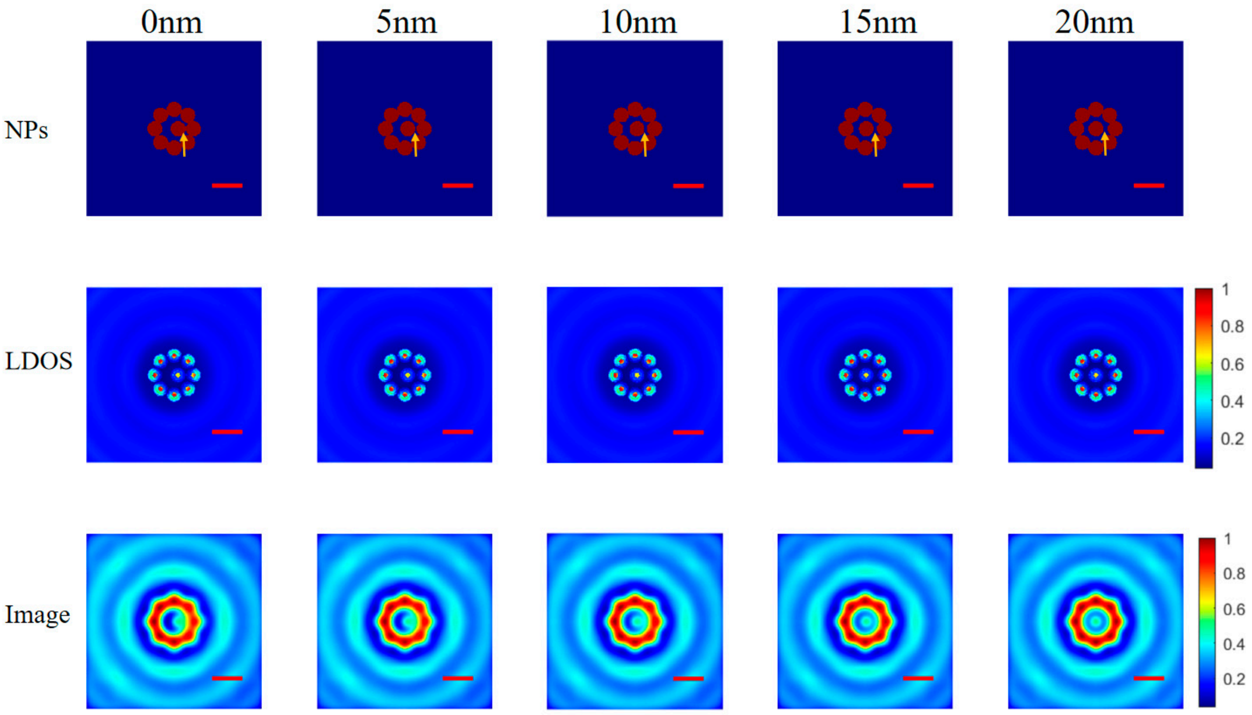

Figure S5. Simulated LDOS (second row) and image (third row) distribution for corresponding AuNPs arrangements (first row) with different gap distance between the central AuNP and right adjacent one. The yellow arrow denotes the gap distance. Scale bars:160 nm.

IV. Influence of Environmental Refractive Index to the LDOS

Different immersion liquid will change the far-field LDOS since the environmental refractive index changes accordingly. Figure S6 shows the far-field mapping of the LDOS of a single NP. It can be seen that the intensity of the LDOS changes in different surrounding materials. In addition, the environmental refractive index will vary the PSF that the FWHM of the far-field mapping of LDOS will also change.

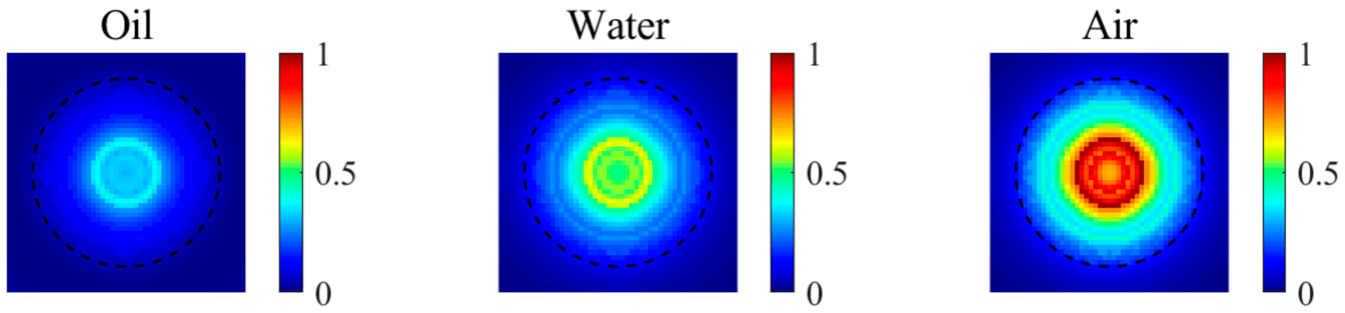

Figure S6. The simulated LDOS of a single AuNP shown in Figure 4a in the immersion materials of oil, water, and air, respectively. The back circle indicates the projection boundary of AuNP with the diameter of 80 nm.
